# Gestational Changes in Exercise Capacity in Uncomplicated Singleton Pregnancies

**DOI:** 10.1016/j.jacadv.2026.102791

**Published:** 2026-06-17

**Authors:** Erin R. Dwyer, Laurie A. Soine, Elizabeth Bayley, Catherine M. Albright, Jonathan Buber

**Affiliations:** a University of Washington Division of Cardiology, Seattle, Washington, USA; b University of Washington Department of Obstetrics and Gynecology, Seattle, Washington, USA

**Keywords:** cardiology, cardiopulmonary, exercise, pregnancy

## Abstract

**Background:**

Pregnancy is associated with well-characterized changes to the cardiopulmonary systems, yet few studies have quantified exercise capacity across trimesters in healthy pregnancies.

**Objectives:**

This study aimed to prospectively assess exercise capacity throughout pregnancy via cardiopulmonary exercise testing (CPET).

**Methods:**

Healthy pregnant volunteers underwent ramp-protocol cycle-ergometer CPET in the 1st and 3rd trimesters. Volunteers were followed through delivery to record delivery mode, gestational age at delivery, fetal birth weight, and pregnancy complications. The mean CPET measures were summarized and compared between the 1st and 3rd trimesters with paired t-tests.

**Results:**

A total of 25 pregnant volunteers participated, with a mean age of 31.4 years (SD 3.8). All volunteers had normal baseline echocardiograms (median left ventricular ejection fraction 60%, IQR: 60%-62%). Between the 1st and 3rd trimester, there were no statistically significant differences in the mean peak oxygen consumption (1,804 vs 1,894 mL/min, *P* = 0.58), mean maximal oxygen uptake at the anaerobic threshold (1,236 vs 1,227 mL/min, *P* = 0.95), mean O_2_ pulse (11.0 vs 11.7 mL/beat, *P* = 0.39), and mean ventilatory efficiency (minute ventilation/volume of carbon dioxide produced and exhaled by the body per minute slope 31 vs 33, *P* = 0.28). The mean resting and peak heart rate increased significantly (*P* < 0.01), whereas the mean maximal voluntary ventilation (105 vs 98, *P* = 0.004) and hemoglobin concentration (13.0 vs 11.8, *P* < 0.01) decreased. No complications occurred during the CPETs or during labor and delivery.

**Conclusions:**

Despite the expected changes in hemoglobin and lung capacity, exercise capacity remained unchanged between the 1st and 3rd trimesters of pregnancy in healthy volunteers.

Pregnancy imposes dynamic physiological changes on the cardiovascular, respiratory, and musculoskeletal systems to meet the metabolic demands of the growing fetus. In singleton pregnancies, cardiac output rises by up to 50%, heart rate and stroke volume increase, systemic vascular resistance declines by about 30%, and blood volume expands by 60 to 70%.^1^^,^^2^ Although these changes are well described at rest, less is known about changes in exercise physiology during pregnancy. Understanding maternal cardiovascular performance during exercise is essential, as physical activity represents both a natural stressor and a potential modifier of pregnancy outcomes.

Exercise testing during pregnancy provides a unique opportunity to quantify maternal cardiac and respiratory reserves, elucidate hemodynamic adaptations, and identify abnormal responses that may predict complications such as preeclampsia, gestational hypertension, or cardiac decompensation.^3^^,^^4^ Moreover, graded exercise testing with cardiopulmonary monitoring offers objective insight into oxygen delivery, ventilatory efficiency, and myocardial workload parameters that cannot be captured by resting assessment alone. Despite this potential, exercise testing in pregnancy remains underutilized, largely due to safety concerns, limited normative data, and lack of standardized interpretation frameworks.^5^

Describing the physiologic responses to exercise across gestation using cardiopulmonary exercise testing (CPET) could therefore enhance the understanding of pregnancy-specific exercise physiology, provide reference values for CPET interpretation in pregnant participants, and inform public health recommendations. For women with known cardiovascular disease, CPET may guide risk stratification, antenatal counseling, and delivery planning. For healthy pregnancies, CPET may clarify how habitual fitness and physical activity influence maternal adaptation and fetal well-being.

The aim of this study was to provide the first prospective evaluation of maternal cardiovascular and ventilatory responses to graded exercise during the 1st and 3rd trimesters of pregnancy and characterize normal adaptation patterns.

## Methods

We conducted a prospective study enrolling healthy pregnant volunteers aged ≥18 years. All participants underwent a baseline transthoracic echocardiogram (TTE) during the 1st trimester that was read by an expert Cardiologist, followed by 2 standardized cycle-ergometer CPETs performed during the 1st and 3rd trimesters of pregnancy. Volunteers with known chronic muscular, metabolic, pulmonary, or cardiac disease or symptoms suggesting an underlying medical condition were excluded from participation in the study. If a cardiac abnormality was diagnosed on the screening TTE, the participant was excluded from the study and scheduled for a Cardiology clinic appointment. TTEs were performed using Philips CVX Epiq and GE Healthcare Vivid E95 ultrasound machines. Before the first CPET, participants completed a brief questionnaire assessing habitual physical activity, adapted from the International Physical Activity Questionnaire.^6^

CPETs were performed in a nonfasting state using an upright cycle ergometer. For standardization, a ramp protocol of 15 was used for all participants. At the beginning of each test, the metabolic cart was calibrated, the participant was connected to the metabolic cart, and baseline metabolic and hemodynamic data was collected for 3 minutes. Exercise was then performed with cardiopulmonary monitoring, including breath-to-breath O_2_ and CO_2_ measurement (Ultima TM CardioO2 gas exchange analysis system; MGC diagnostics). Heart rate and rhythm were documented via continuous 12-lead electrocardiogram recording. Blood pressure was recorded every 2 minutes and at peak exercise. The symptom that limited maximal exercise testing was recorded. Peak exercise parameters included peak heart rate, VO_2_max (both absolute values and indexed to the participant weight in kilograms), % predicted VO_2_max, respiratory equivalent ratio, peak O_2_ pulse (defined as the VO_2_max divided by the peak heart rate) and the slope of ventilation per liter of expired carbon dioxide measured over the entire CPET (minute ventilation/volume of carbon dioxide produced and exhaled by the body per minute slope).

The mean CPET measures and corresponding SDs, including baseline pulmonary function testing, were summarized descriptively in the 1st and 3rd trimesters. CPET and pulmonary function test values were compared between the 1st and 3rd trimesters with paired t-tests. A threshold of 0.05 was used to determine statistical significance. All analyses were performed in R (version 4.2).^7^

The study was reviewed and approved by the University of Washington Office of Research Human Subjects Division. Informed consent of all participants was obtained.

## Results

### Demographics

Twenty-five pregnant volunteers were prospectively enrolled. The mean age at 1st trimester CPET was 31.4 ± 3.8 years and mean body mass index was 23.3 ± 4.4 kg/m^2^. Most participants self-identified as White (80%) or Asian (16%), all had private insurance, and most were nulliparous (80%). All echocardiograms performed in the 1st trimester before the first cardiopulmonary exercise test confirmed normal cardiac and great vessel structure and diameters, normal biventricular function (mean left ventricular ejection fraction of 61% ± 3.4%), normal diastolic filling pattern, no more than trace valvular abnormality, and normal estimated central venous and systolic pulmonary arterial pressures (Table 1). Baseline exercise engagement surveys suggested that most participants were relatively sedentary at the time of the 1st trimester CPET. When asked “In a typical week, on how many days do you do moderate-intensity sports, fitness, or recreational activities?”, the median answer was 0 days, with an IQR of 0 to 3 (Supplemental Table 1).

**Table 1:** Demographic and Baseline Echocardiogram Data (N = 25)

Age, y	31.4 ± 3.8
Race	
White	20 (80)
Black	1 (4)
Asian	4 (16)
Insurance	
Private	25 (100)
Gravity	
1	17 (68)
2	6 (24)
3	2 (8)
Parity	
0	20 (80)
1	5 (20)
BMI, kg/m^2^	23.3 ± 4.4
Baseline echocardiogram	
LVEF	61.0 ± 3.4
RV dysfunction	0 (0)
Estimated PASP	22.0 ± 3.3
Baseline systolic blood pressure	115 (9.6)

Values are mean ± SD or n (%).

BMI = body mass index; LVEF = left ventricular ejection fraction; PASP = pulmonary arterial systolic pressure; RV = right ventricle.

### Cardiopulmonary exercise testing

The mean gestational ages at the time of the 1st and 3rd trimester CPETs were 12 ± 1.5 and 35 ± 1.9 weeks, respectively. The mean duration between CPETs was 23 ± 2.4 weeks. Hemoglobin, obtained within 1 month of each CPET were available for all participants and demonstrated an expected decline (1st trimester: 13 ± 0.8 vs 3rd trimester: 12 ± 0.7; *P* < 0.001) with concomitant lower hematocrits.

Exercise parameters are presented in Table 2. The mean resting heart rate, measured before the beginning of each CPET, increased by 9 beats per minute (beats/min) between the 1st and 3rd trimesters (76 ± 7 beats/min vs 85 ± 12 beats/min; *P* < 0.01), and the mean peak heart rate increased by 7 beats/min (137 ± 10 beats/min vs 144 ± 29 beats/min, *P* < 0.01). Resting and peak systolic blood pressures did not significantly differ between the 1st and the 3rd trimesters (resting: 113 ± 10 mm Hg vs 113 ± 12 mm Hg; peak: 170 ± 18 mm Hg vs 175 ± 18 mm Hg, respectively). Baseline spirometry was obtained before each CPET. The forced expiratory volume in 1 second and forced vital capacities were similar between trimesters; however, there was a notable decline in the mean maximal voluntary ventilation (106 ± 10 mL vs 98 ± 10 mL, *P* = 0.004).

**Table 2:** Cardiopulmonary Exercise Testing Measures in 1st and 3rd Trimesters of Pregnancy

Measurements Taken at Time of CPET	1st Trimester		3rd Trimester		*P* Value^a^
	Mean	SD	Mean	SD	
Hemoglobin (g/dL)	13	0.8	12	0.7	**<0.001**
Exercise duration (min)	15.9	4.2	16	3.9	0.9301
Resting systolic blood pressure (mm Hg)	115	9.6	113	11.8	0.5408
Peak systolic blood pressure (mm Hg)	170	17.5	175	17.6	0.1931
Resting diastolic blood pressure (mm Hg)	68	7.7	69	8.8	0.7223
Peak diastolic blood pressure (mm Hg)	74	9.5	75	10.5	0.7339
Resting heart rate (beats/min)	76	7.3	85	12.1	**0.0048**
Peak heart rate (beats/min)	137	10	144	29	**<0.001**
Watts	166	31.7	172	39.1	0.5102
Resting oxygen consumption (mL/min)	249	10.6	342	11.3	**<0.001**
Peak oxygen consumption (nonindexed, mL/min)	1804	471.9	1894	534.8	0.5869
Change in oxygen consumption (mL/min)	1,555	462.7	1,552	533.9	0.986
% predicted peak oxygen consumption	112	27.8	109	29.6	0.6845
O_2_ pulse absolute (mL/beat)	11	2.9	12	3.3	0.3886
% predicted O_2_ pulse^b^	112	27.8	114	31.6	0.8686
Heart rate response (%)	91	6	100	8.5	**<0.001**
VO_2_ at anabolic threshold (mL/min)	1,236	491.4	1,227	518.8	0.9536
% VO_2_ at anabolic threshold^b^	67	13.6	63	13	0.2362
Maximal voluntary ventilation (L/min)	106	9.8	98	10	**0.004**
Respiratory exchange ratio	1.3	0.13	1.3	10.5	0.196
Minute ventilation at peak effort (L/min)	88	10	98	12	**0.004**
VE/VCO_2_ slope	31	3.8	33	3.9	0.277
PFTs					
FEV1 (L)	97.2	9.8	95.9	10.7	0.6439
FVC (L)	101.4	8.1	97.5	8.9	0.112
FEV1/FVC	97.2	9.8	95.9	10.7	0.659

**Bold** values indicate statistical significance of *P*-value <0.05.

FEV1 = forced expiratory volume in first second; FVC = forced vital capacity; PFT = pulmonary function testing; VE = ventilatory efficiency; VCO_2_ = volume of carbon dioxide produced and exhaled by the body per minute; VO_2_ = maximal oxygen uptake.

a *P* values generated from paired t-tests.

b Generated from reference values.^8^^,^^13^

**Figure 1:**
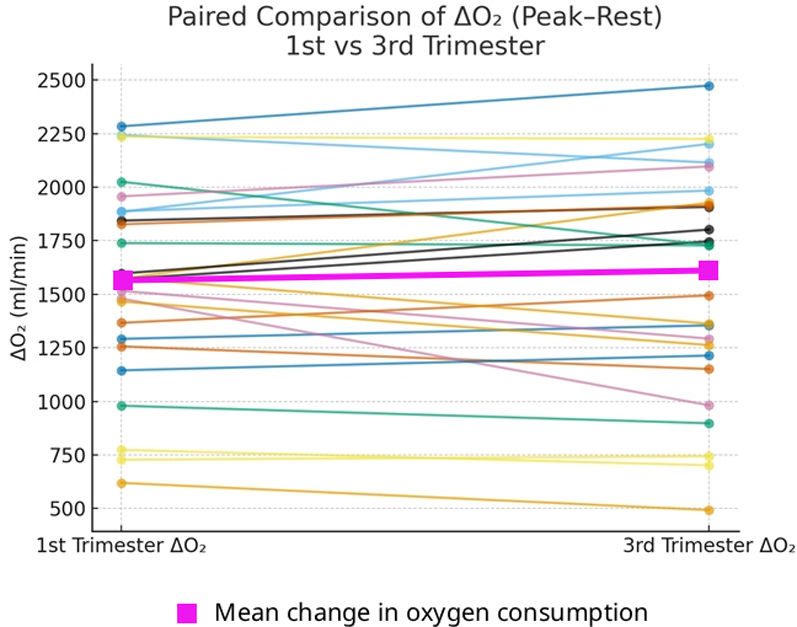
**Changes in Peak VO_2_ Between 1st and 3rd Trimesters** This figure displays the change in oxygen consumption (single value representing difference between peak and resting oxygen consumption from the same cardiopulmonary exercise test) among each individual participant between the 1st and 3rd trimesters. VO_2_ = maximal oxygen uptake.

The average weight gain between the 2 CPETs was 17 ± 4 kg. With that, the *resting* oxygen consumption significantly increased from the 1st to the 3rd trimester (249 ± 11 mL/min vs 342 ± 11 mL/min, *P* < 0.001), whereas the *peak* oxygen consumption did not significantly change (1,804 ± 472 mL/min vs 1,894 ± 535 mL/min, *P* = 0.587). These differences did not translate into an overall significant difference in the resting-to-peak increase in oxygen consumption during exercise between the 1st and 3rd trimesters (increase of 1,555 ± 463 mL/min vs 1,552 ± 534 mL/min, *P* = 0.98) (Figure 1).

**Figure 2:**
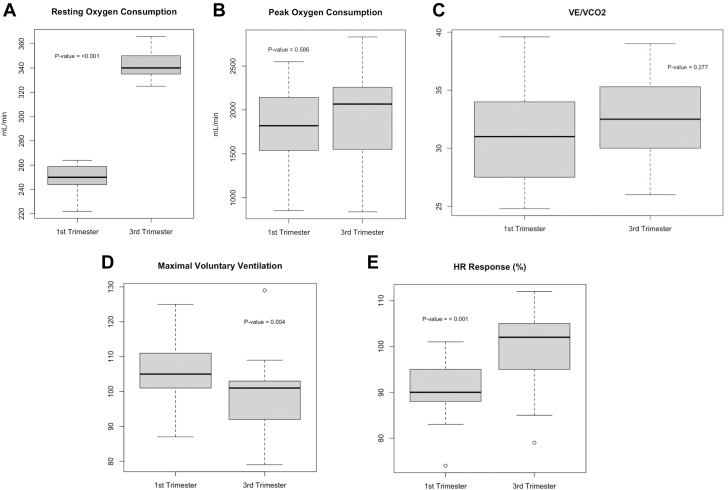
Mean Values for Select CPET Measures in the 1st and 3rd Trimesters (A) mean resting oxygen consumption in the 1st and 3rd trimester cardiopulmonary exercise testing. Boxplot displaying the difference in mean resting oxygen consumption between the 1st and 3rd trimester CPET. The *P* value was generated with a paired t-test and suggests a significant difference between the mean resting oxygen consumption between the 1st and 3rd trimester CPET. (B) Mean peak oxygen consumption in the 1st and 3rd trimester cardiopulmonary exercise testing. Boxplot displaying the difference in mean peak oxygen consumption between the 1st and 3rd trimester CPET. The *P* value was generated with a paired t-test and suggests a nonsignificant difference between mean peak oxygen consumption between the 1st and 3rd trimester CPET. (C) Mean VE/VCO_2_ in the 1st and 3rd trimester cardiopulmonary exercise testing. Boxplot displaying the difference in mean VE/VCO_2_ between the 1st and 3rd trimester CPET. The *P* value was generated with a paired t-test and suggests a nonsignificant difference between mean VE/VCO_2_ between the 1st and 3rd trimester CPET. (D) Mean maximal voluntary ventilation in the 1st and 3rd trimester cardiopulmonary exercise testing. Boxplot displaying the difference in mean Maximal Voluntary Ventilation between the 1st and 3rd trimester CPET. The *P* value was generated with a paired t-test and suggests a nonsignificant difference between mean Maximal Voluntary Ventilation between the 1st and 3rd trimester CPET. (E) Mean heart rate response in the 1st and 3rd trimester cardiopulmonary exercise testing. Boxplot displaying the difference in mean Heart Rate Response between the 1st and 3rd trimester CPET. The *P* value was generated with a paired t-test and suggests a nonsignificant difference between mean Heart Rate Response between the 1st and 3rd trimester CPET. HR = heart rate; VCO_2_ = volume of carbon dioxide produced and exhaled by the body per minute; VE = ventilatory efficiency.

The maximal oxygen uptake (VO_2_) at the anaerobic threshold (1,236 ± 491 mL/min vs 1,227 ± 519 mL/min, *P* = 0.95), the O_2_ pulse (11 ± 3 mL/beat vs 12 + 3 mL/beat, *P* = 0.39), and the ventilatory efficiency (quantified by the minute ventilation/volume of carbon dioxide produced and exhaled by the body per minute slope: 31 ± 4 vs 33 ± 4, *P* = 0.28) were similar between the 1st and 3rd trimesters (Figures 2A to 2E).

### CPET tolerability and pregnancy outcomes

All participants were able to complete both CPETs without cardiac symptoms, and no obstetric complications occurred during or after the studies. Two participants (2/25, 8%) were diagnosed with gestational hypertension during their pregnancy and none were diagnosed with gestational diabetes. All participants delivered at full term (median gestational age 39 weeks [IQR: 38-41 weeks]) and most delivered vaginally (18/25, 72%). The mean fetal birth weight was 3,226 ± 247 g. Three fetuses had intrauterine growth restriction, 2 of which were born to the participants who developed gestational hypertension. Although the small sample size precluded formal statistical analysis, the peak systolic blood pressures during the first trimester CPETs were notably higher in the 2 participants who later developed gestational hypertension than in the rest of the cohort (198 mm Hg and 203 mm Hg).

## Discussion

Normal pregnancy is characterized by progressive hemodynamic adaptations that begin early and include expansion of plasma volume and a decline in systemic vascular resistance, resulting in a reduction in blood pressure during the first trimester. There is an early rise in the cardiac output by 30% to 50%, which is initially driven by increased stroke volume and later by a physiologic increase in heart rate by 10 to 20 beats/min. Systemic vascular resistance falls by up to 30% to 50%, reaching its nadir in mid-pregnancy, at which time blood pressure is typically lowest. As gestation progresses, blood pressure gradually returns toward pre-pregnancy levels and mechanical compression of the inferior vena cava by the gravid uterus may reduce venous return and cardiac output.

Although these changes have been repeatedly validated and are well acknowledged, their effect on exercise physiology has rarely been assessed. In this prospective study, we aimed to provide the first detailed characterization of normal physiological adaptations to exercise during pregnancy in a small cohort of pregnant patients without known medical conditions. A key finding of this study is that maximal effort cycle-ergometer–based CPET performed during the 1st or 3rd trimester appeared safe and well tolerated in healthy volunteers with singleton pregnancies as no cardiovascular, pulmonary, or obstetric complications were observed among study participants during the CPETs or labor and delivery. In the context of the normal physiological adaptations of pregnancy, several findings in this study—such as higher resting and peak heart rates and lower hemoglobin levels—were anticipated. Although subtle changes in forced expiratory volume in 1 second and forced vital capacities during pregnancy have been reported previously, no such changes were observed in our cohort. In contrast, the higher peak minute ventilation during exercise and the lower maximal voluntary ventilation demonstrate the physiological hyperventilation of pregnancy.

Although heart rate increased progressively over the course of pregnancy, peak VO_2_ remained unchanged, accompanied by a stable O_2_ pulse, indicating preserved stroke volume and peripheral oxygen extraction between the 1st and 3rd trimesters. One plausible explanation for this observation is the reduction in oxygen delivery associated with the expected anemia of pregnancy observed in our cohort, which may have limited further increases in VO_2_ despite the rise in heart rate (Central Illustration).

**Central Illustration:**
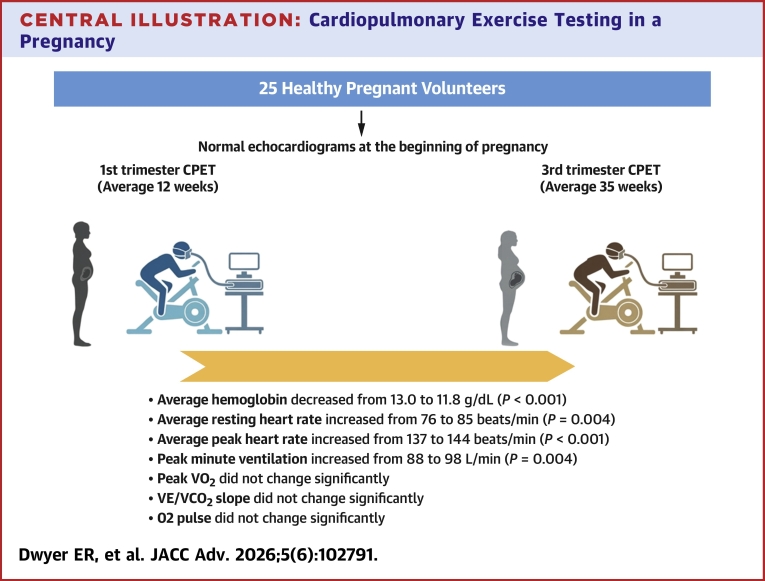
**Cardiopulmonary Exercise Testing in a Pregnancy** 25 healthy volunteers with singleton pregnancies were recruited during the 1st trimester to undergo cardiopulmonary exercise testing at the time of recruitment and again in the 3rd trimester. No complications occurred, and exercise capacity as estimated by CPET was similar in the 1st and 3rd trimester. CPET = cardiopulmonary exercise testing; VCO_2_ = volume of carbon dioxide produced and exhaled by the body per minute; VE = minute ventilation; VO_2_ = maximal oxygen uptake.

Other exercise parameters remained largely unchanged as pregnancy progressed, including ventilatory efficiency, despite higher peak minute ventilation and an augmented blood pressure response to exercise. Notably, the 2 participants who subsequently developed gestational hypertension exhibited substantially higher peak systolic blood pressures during exercise compared with the remainder of the cohort. Consistent with this observation, our group has previously demonstrated that hypertensive responses to exercise are strongly associated with the future development of gestational hypertension in patients with repaired coarctation of the aorta. Further evaluation of this hypothesis in larger cohorts without underlying cardiac disease is warranted.^3^

Although little data exist on tolerability, safety, and prognostic capability of CPET in pregnancy, other forms of exercise stress testing have proven to be good predictors of the pregnancy tolerance and functional capacity, particularly in women with known or suspected heart disease. The 6-minute walk test, submaximal cycle ergometry, and treadmill testing are the most studied methods. In healthy, term, nulliparous women, the mean distance walked on a 6-minute walk test was 488 m (95% reference interval 302-674 m), with a mean heart rate increase of 12 beats/min and median recovery time of 5 minutes.^9^ The more limited data on submaximal cycle ergometry and treadmill tests showed that these can provide objective assessment of maternal functional capacity and can improve identification of exercise-induced arrhythmias.^10^^,^^11^

As our study did not include a nonpregnant control group and we lacked pre-pregnancy exercise data for our cohort to assess whether early changes in exercise capacity already take place in the 1st trimester, we compared our results to reference values from a recently published study of cardiopulmonary exercise testing in a large American registry of healthy individuals. In that analysis, peak exercise capacity in age-matched, nonpregnant participants who exercised on a cycle ergometer was slightly lower than in the 1st trimester participants in our study (1,740 mL/min vs 1,804 mL/min), although both groups reached similar maximal effort (nonpregnant, aged-matched: mean RER 1.16 + 0.09, our study 1st trimester: respiratory exchange ratio of 1.13 + 0.13 in our cohort). However, this comparison is subject to many limitations.^12^

CPET is a well-established modality for perioperative risk stratification and for predicting morbidity and mortality.^5^^˒^^9^ However, CPET has been underutilized in pregnant populations due to safety concerns, resulting in a paucity of baseline data for comparison.^9^ As such, future research should aim to develop reference data for CPET measures during pregnancy in a large, diverse cohort. The safety demonstrated in our study should encourage further research and broader implementation of CPET in pregnancy, where it has the potential to serve as a valuable tool within the expanding field of cardio-obstetrics. Specifically, CPET may facilitate identification of underlying cardiopulmonary disease, prediction of pregnancy-related complications and future cardiovascular risk, predelivery risk stratification, and clarification of exercise tolerance and limitations in higher-risk pregnant individuals.^5^ Furthermore, our findings provide pilot normative reference data from healthy singleton gestations that can serve as a comparator as CPET use expands to pregnant individuals with cardiac, pulmonary, or metabolic disease, as well as those with multifetal gestations.^13^

### Study limitations

The strengths of our study include its prospective design, no loss to follow-up, and inclusion of a baseline echocardiogram in all participants. One inherent limitation of this study is the lack of pre-pregnancy exercise data of the study cohort, as all participants were recruited while already pregnant. Another limitation is the small, minimally diverse cohort included in our study. Only healthy, singleton gestations were included to assess CPET safety in an appropriate population and generate comparison data for further CPET use; this limits the generalizability to a large proportion of the United States pregnant population. Using less costly screening techniques than an echocardiogram, such as brain natriuretic peptide levels and an electrocardiogram could be a viable option to be considered in larger prospective studies.

## Conclusions

In a cohort of healthy volunteers carrying a singleton pregnancy, we show that maximal effort studies performed on a cycle ergometer during the first and the third trimesters were well tolerated without complications. Although heart rates and minute ventilation increased and the hemoglobin levels decreased between the first and the third trimesters, blood pressure response and exercise tolerance remained stable, with similar peak VO_2_, ventilatory efficiency, and oxygen pulse values.

## Funding support and author disclosures

Funding for this study was provided by the 10.13039/100005644Alpha Phi Foundation. The authors have reported that they have no relationships relevant to the contents of this paper to disclose.
