# Supplementary material for: Gestational Changes in Exercise Capacity in Uncomplicated Singleton Pregnancies
Source: JACC Adv. 2026 Jun 17;5(6):102791. doi: 10.1016/j.jacadv.2026.102791 (PMC13309298; doi:10.1016/j.jacadv.2026.102791)
Supplement: Supplemental_Table [file mmc1.docx]

Supplemental Table 1

|  | N | % |
| --- | --- | --- |
| Does your work involve vigorous-intensity activity that causes large increases in breathing or heart rate for at least 10 minutes continuously? | 0 | 0 |
| Does your work involve moderate-intensity activity, that causes small increases in breathing or heart rate such as brisk walking for at least 10 minutes continuously? | 12 | 48 |
| In a typical week, on how many days do you do moderate-intensity activities as part of your work? | 0 (median) | 4 (IQR) |
| How much time do you spend doing moderate-intensity activities at work on a typical day (in minutes)? | 0 (median) | 40 (IQR) |
| Do you walk or use a bicycle (pedal cycle) for at least 10 minutes continuously to get to and from places? | 7 | 28 |
| In a typical week, on how many days do you walk or bicycle for at least 10 minutes continuously to get to and from places? | 0 (median) | 3 (IQR) |
| How much time do you spend walking or bicycling for travel on a typical day (in minutes)? | 0 (median) | 20 (IQR) |
| Do you do any vigorous-intensity sports, fitness or recreational activities that cause large increases in breathing or heart rate for at least 10 minutes continuously? 0=no, 1=yes | 12 | 48 |
| In a typical week, on how many days do you do vigorous-intensity sports, fitness or recreational activities? | 0 (median) | 4 (IQR) |
| How much time do you spend doing vigorous-intensity sports, fitness or recreational activities on a typical day (in minutes)? | 0 (median) | 60 (IQR) |
| Do you do any moderate-intensity sports, fitness or recreational activities that causes a small increase in breathing or heart rate for at least 10 minutes continuously? 0-no, 1=yes | 8 | 32 |
| In a typical week, on how many days do you do moderate-intensity sports, fitness or recreational activities? | 0 (median) | 3 (IQR) |
| How much time do you spend doing moderate-intensity sports, fitness or recreational (leisure) activities on a typical day (in minutes)? | 0 (median) | 30 (IQR) |
| How much time do you usually spend sitting or reclining on a typical day (in minutes)? | 210 (median) | 240 (IQR) |
